# The role of context in strategic human resource management within private hospitals in Ethiopia, compared to public hospitals

**DOI:** 10.3389/frhs.2026.1740353

**Published:** 2026-02-19

**Authors:** Philipos Petros Gile, Joris van de Klundert, Martina Buljac-Samardžić

**Affiliations:** 1 Erasmus School of Health Policy & Management, Erasmus University Rotterdam, The Netherlands & Higher Education Institutions’ Partnership, Addis Ababa, Ethiopia; 2 School of Business, Universidad Adolfo Ibanez, Santiago de Chile, Chile & Erasmus University Rotterdam, Erasmus School of Health Policy & Management, Rotterdam, Netherlands; 3 Erasmus School of Health Policy & Management, Erasmus University Rotterdam, Rotterdam, Netherlands

**Keywords:** context, Ethiopia, policies, private hospitals, regulations, strategic human resource management

## Abstract

**Introduction:**

Private hospitals in Ethiopia complement the resource constrained public hospitals. Health policy reforms have promoted further expansion of the private sector, despite a rigid policy that provide little room for strategic human resource management(SHRM). Consequently, private hospitals encounter challenges to live up to their potential. This study investigates the contextual mechanisms influencing SHRM in private for-profit (PFP) and not-for-profit (PNFP) hospitals, in comparison to public hospitals.

**Materials and methods:**

A qualitative approach was employed, utilizing structured interviews with CEOs, administrators, and HR managers from ten private hospitals. To make the comparison between private and public hospitals possible, the same protocols of the companion studies in public hospitals was followed. Thematic analysis through deductive coding based on the Contextual SHRM framework was conducted, using ATLAS.ti 8 software.

**Results:**

Our findings reveal that PNFP hospitals benefit from constructive government engagement and experiencing less coercive pressure, which appears to enable them to address underserved populations and mitigate public hospital capacity issues. In contrast, PFP hospitals perceive regulations as rigid and constraining. Though PFP hospitals compete for patients, they employ highly skilled workforce as a means towards delivering higher quality and valuable services to justify the higher price charged to patients.

**Conclusions:**

Our study shows that stringent governmental policies/regulations exert pressure on SHRM of PFP hospitals. In contrast, PNFP hospitals perceive regulations as supportive. This condition is different from public hospitals which lack rooms to maneuver for crafting SHRM. Future research agenda are called for engaging employees for valuable insights into SHRM practices and performance.

## Introduction

The importance of context for strategic human resource management (SHRM) has received explicit interest in the last two decades (1–3). This interest into contextual factors extends to SHRM in hospitals, and more specifically to the impact of ownership types within health systems (4–7). Various authors have for instance studied how market regulation and differences in regulation depending on ownership type impact SHRM in high income countries (8, 9). Hospital ownership types are especially relevant in low- and middle-income countries (LMICs) such as Ethiopia, in which private providers often complement the public hospitals operating within resource constraint health systems (5, 10, 11). In such contexts, private-for-profit (PFP) and private non-for-profit (PNFP) hospitals can alleviate the burden on the public systems and improve access to care (12).

In Ethiopia, the private sector provides a wide range of health facilities offering diverse health services and products, across all regions, cities and levels of health care system from primary- to specialized tertiary care (13, 14). It includes PNFP facilities by missionaries, civil society organizations, and PFP hospitals. The PFP facilities mostly serve the high- and middle-income groups, while the PNFP providers, together with the public ones, serve low-income groups (15–17). The private sector has grown rapidly in size and significance in recent years (18–23), making it difficult to determine the exact number of facilities in general or specified to type (e.g., hospital). However, estimates suggest that private health service providers account for 27% of the approximately 28.000 Ethiopian healthcare facilities (19, 21, 24).

The recent rapid expansion of private providers reflects the growing demand for health services and underscores the ongoing challenges faced by the Ministry of Health in governing accessibility of high-quality health services (15, 22). Recent health policy reforms have promoted further expansion of the private sector and enabled them to play a more significant role in health service provision (13, 17, 23). The Ethiopian government recognizes the challenges and regulatory barriers facing the private sector and is actively pursuing additional reforms to address these issues (24–28). For instance, the Human Resource Information System (HRIS) was introduced in 2009 to better govern human resources together with the private sector (24, 25). Still, further reforms and particularly deregulations have been argued to be crucial, as the tight governmental regulation might exacerbate rather than resolve the Ethiopian health human resource management crisis (10, 11, 14, 23, 26, 27). However, there is little scientific understanding of SHRM in relation to hospital ownership type in Ethiopia, nor in Sub-Saharan Africa (SSA).

Our previous work on public hospitals in SSA and Ethiopia identified that regulations are often perceived as strict and to leave limited leeway for crafting SHRM (10, 11, 28). These studies indicated that government regulations and budget limitations appeared to hinder public hospitals to address persistent SHRM challenges, in particular in relation to fair compensations, attracting and retaining personnel, and better working conditions. The latter are regarded as the core drivers of the nationwide strikes in state-owned hospitals in the spring of 2025.

In addition, the perceived long standing strong governmental control of SHRM in Ethiopian public hospitals, characterized by the top-down enforcement of stringent regulations and, HR policies, negatively related to staff wellbeing and engagement (10, 11). At the same time, these studies indicated that this difficult context triggered some public hospitals to invent innovative SHRM practices in this context.

Regarding private hospitals, there is evidence from various contexts that SHRM practices can contribute to better health services, and patient and employee outcomes, in comparison to public hospitals (29–34). Physicians in private hospitals have been reported to be significantly more satisfied (compared to nurses) because of preferential incentives to motivate physicians (29, 30). Studies also indicated job satisfaction to be positively associated with patient satisfaction (10, 11, 31–34). In several Sub-Sharan Africa contexts, private hospitals have been reported to provide better health services than public hospitals [e.g., (12, 29, 35–42)]. This has been associated with better working conditions, fair compensations, more effective incentive schemes to promote and reward highly motivated employees and addressing underperformers (12). More generally, there is evidence suggesting that PFP hospitals are more innovative in addressing HRM issues such as motivation, incentives, satisfaction, retention (12, 15–17, 43, 44), and the improvement of subsequent performance outcomes including quality of health services (4, 5, 10)^1^. Thereby, various studies report that PNFP hospitals tend to perform better than private for-profit (PFP) hospitals and public hospitals due to a combination of government stewardship benefits and non-profit motives (5, 28, 45–48). However, the relationships between SHRM with patient and employee outcomes are not well researched and documented in private hospitals for SSA in general and Ethiopia in specific (11, 12, 37, 41, 42).

In this study, we hypothesize that the less rigid regulation of private hospitals enables their SHRM to advance beyond SHRM practices in public hospitals. More specifically, we posit that private hospitals are more effective at attracting and retaining an adequately skilled workforce, as a mechanism towards providing better staff well-being and higher quality services. The latter is necessary for private hospitals providing services against higher charges than the (almost) free of charge public hospitals. However, these hypotheses have not been well researched, leaving a research gap regarding the role of context in SHRM within private hospitals and how this compares to public hospitals.

**Figure 1:**
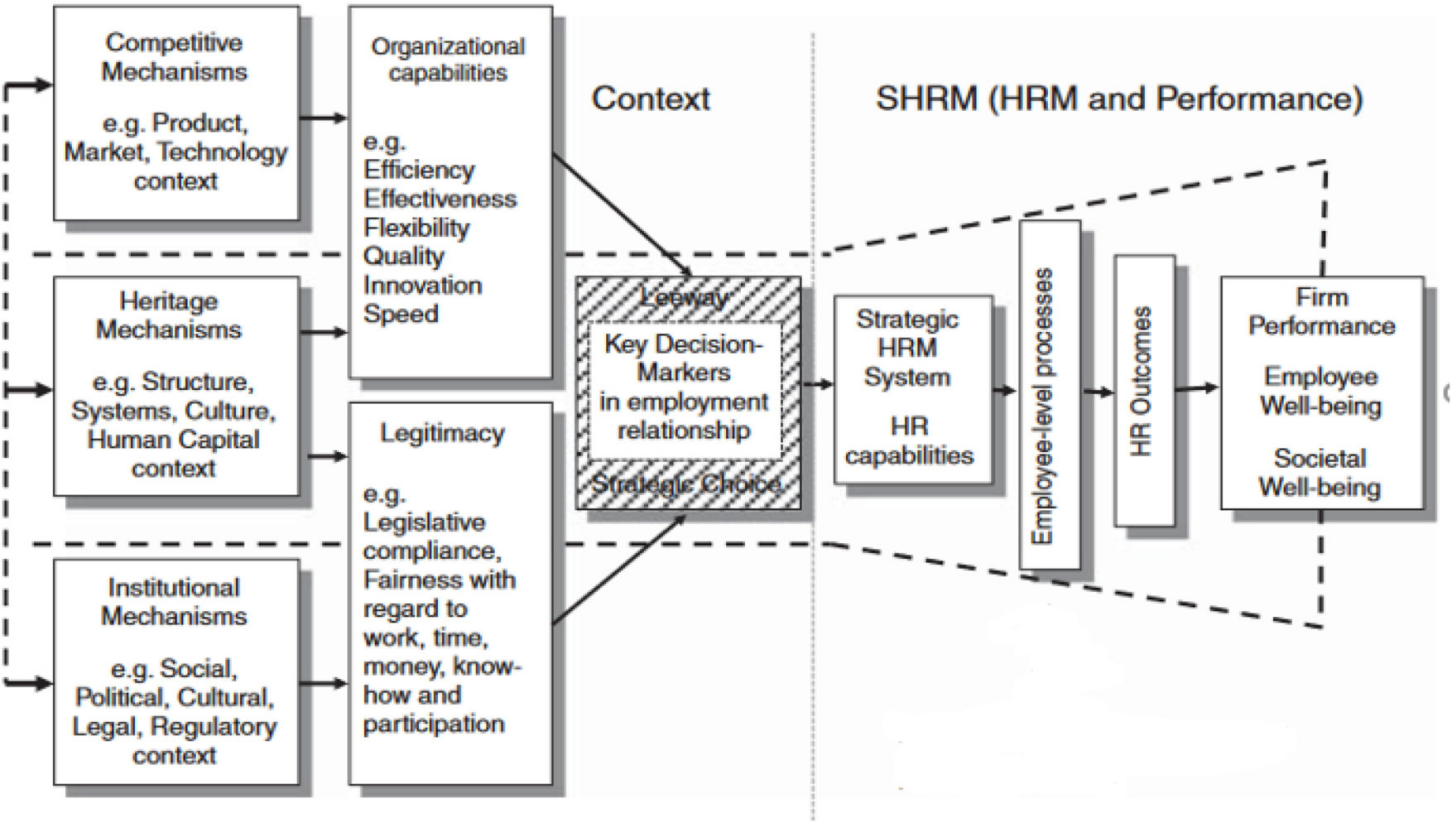
Contextual SHRM framework (49).

Our research thus aims to understand how contextual mechanism influence HRM in private (both for- profit and not-for-profit) hospitals in Ethiopia and subsequently relate to employee and patient outcomes, in comparison to previous results obtained for public hospitals (10, 11). In recognition of the strategic impact of contextual mechanisms on HRM (see also Figure 1), our main, comparative, research questions therefore are (1) “Which contextual mechanisms influence strategic HRM and performance in private Ethiopian hospitals—when compared with public hospitals?” and (2) “How do these differ between private and public hospitals?”

## Materials and methods

Because of the qualitative and comparative nature of the research question, we conducted a qualitative study through structured interviews with respondents from private hospitals in Ethiopia, following the same protocols of the companion studies in public hospitals (10, 11). This section therefore follows the logic of the companion study in public hospitals (10, 11).

Numerous studies examining the effects of Strategic Human Resource Management (SHRM) on outcomes have incorporated contextual mechanisms or factors. Paauwe's model offers a contextual SHRM Framework that integrates many of these studies and the models therein along with relevant theoretical perspectives into one framework (49, 50). The resulting Contextual SHRM Framework serves as the foundation for this study (50–52). Specifically, Paauwe's Contextual SHRM Framework consists of four key components that shape SHRM: institutional mechanisms (e.g., Government regulations, laws, and societal norms imposed by external stakeholders), competitive mechanisms (e.g., market forces influencing HRM and competition for resources or patients), heritage mechanisms (e.g., historical, cultural and organizational factors shaping HRM practices), and leeway [the degree of flexibility organizations (perceive to) have to craft HRM policies and discretion available to managers to maneuver within these constraints] (10, 11, 49).This framework has been adopted by various authors [e.g., 10, 11, 52, 53] to understand and analyse the effects of context on SHRM issues across different sectors, including healthcare (11, 28, 54, 55). The interview protocol is further based in results obtained for private hospitals and a systematic review on SHRM in Sub-Saharan African hospitals (10, 28).

### Data collection

The authors selected 10 private hospitals (3 private not-for profit and 7 private for-profit) by purposive sampling. We aimed to select private hospitals that are representative of the Ethiopian private setting. Authors thus approached a collection of private hospitals that differed in hospital level (i.e., specialized, general, primary), geographical setting (i.e., regional large towns, regional and rural settings, city government of Addis Ababa), and ownership type (Governing body). Table 1 gives details of all the hospitals included for data collection. Data were collected between March and September 2019.

**Table 1:** Private hospital (PNFP and PFP) characteristics selected for data collection.

#	Hospital name	Hospital Level (scope of service)	Geographic Location	Ownership type/Governing body	Established	Beds
1	Hamlin Fistula Hospital	Specialized	Addis Ababa City	Private not-for- profit (Missionary/Trustees)	1974	106
2	Atat Hospital	Primary	SNNP Region	Private not-for-profit(Missionary/Catholic Church)	1970	97
3	Bethel Hospital	General teaching	Addis Ababa	Private for-profit	2000	100
4	Kibru Hospital	Primary	SNNP Region	Private for-profit	2009	30
5	Noah Hospital	Speciality center	Oromia Region	Private for-profit	2016	30
6	Alatyon Hospital	General	SNNP Region	Private for-profit	2011	70
7	Aynalem	Primary	Addis Ababa City	Private for-profit	2015	35
8	Adama Hospital	General teaching	Oromia Region	Private for-profit	2011	205
9	St Luke Hospital	General, Nursing &Midwifery College	Oromia Region	Private not-for-profit (Missionary/Catholic)	2001	200
10	Betezatha Hospital	General	Addis Ababa City	Private for-profit	2003	61

Interviews were carried out with purposefully chosen respondents from various roles within the hospital's administrative and HRM hierarchy, all of whom possess knowledge of and responsibility for the hospital's HRM practices. This approach provided us with a comprehensive insight into the variety of viewpoints on the contextual mechanisms that influence HRM across different departments and levels of hierarchy.

At each hospital we requested to interview the executive board member who was responsible for administration or the head of the HRM department (Table 2 shows the respondents' characteristics). If these respondents were not able to participate or if these positions were not fulfilled within the structure, matrons (who oversee, manage or lead teams of nurses, midwives and support staff in hospitals) were approached. In total, the authors interviewed 10 respondents.

**Table 2:** Respondent characteristics interview.

Hospital/facility	Position	Sex	Educational background	Years in current organization	Years in current position
Hamlin Fistula Hospital HR Head		M	MA, Pub Admin	11 years	2 years
Atat Hospital Administrator		F	MA, Dev’t studies	24years	1o years
Bethel Hospital Administrator		M	BA, Mg’t	20 years	3 years
Kibru Hospital Adm &Finance head		M	BA, Accounting &Finance	7 years	5 years
Noah Hospital Head, HR Dept		F	BA Mg’t	16 years	2 years
Alatyon Hospital Administrator		M	MBA [HR Specialization]	16 years	3 years
Aynalem Hospital Administrator		M	MBA	25 years	3 years
Adama Hospital Administrator		M	MBA	16 years	8 years
St Luke Hospital Matron		F	BSc, Nursing	18 years	8 years
Betazatha Hospital Operations manager		M	MBA; MSc in Economics	11 years	3 months
	Total	10			

The topic list and structured interview guideline (presented in Supplementary Appendix 1 and 2) were jointly constructed by all authors and based on document analysis of the FMOH Health Account and National HRH strategic Plan (23, 26, 27), Paauwe's framework (see Figure 1), and a systematic review on SHRM in sub Saharan Africa hospitals (28, 49).

### Data analysis

Interviews were audio-recorded and transcribed verbatim by the first author after ensuring written consent from all respondents. The transcripts were thematically analysed using ATLAS.ti 8 (10, 11). We first conducted a thematic analysis of the main results from the private hospital interviews. Attention was given to identify and analyze the main (external and internal) contextual factors influencing SHRM and performance in private Ethiopian hospitals. Next, we compared the contextual factors and mechanisms found with the results obtained for private hospitals (10, 11). To this purpose, the original data collected from 19 respondents from public hospitals and the resulting data analysis were reconsidered, specifically looking for findings on factors identified for the private hospitals. Vice versa, we reexamined our data and analysis for private hospitals based on the findings for public hospitals. (see Table 3).

**Table 3:** Summary of SHRM model wise comparative analysis of findings on the mechanisms influencing SHRM in the private hospitals and how this differs from the public hospitals in Ethiopia .

Analysis/elaboration points	(1) Institutional mechanisms	(2) Competitive mechanisms	(3) Heritage mechanisms	(4) Leeway for crafting SHRM	(5) SHRM system, HRM outcomes and influence on performance
findings from private hospitals that are comparable to findings for public hospitals	• tight/control-oriented policy and regulatory framework impacting SHRM • impact of rigid standardization policy in limiting hospital level scope of services and HRM resources • partial compliance with regulatory requirements	• no major comparable findings	• training and mentoring to retain professionals (PNFP hospitals) • build on past government regulations to shape current HRM practices in both PFP and PNFP hospitals	• leeway for innovative non-financial incentive arrangements	• regulatory and health reform frameworks guide HRM and hospital management
findings for private hospitals different from findings for public hospitals	• international non profits have SHRM policies according to international standards in place • noncompliance with mandatory annual employee surveys (especially for profit hospitals)	• able to attract necessary workforce	• leadership is perceived as competent, to be supportive in engaging staff, creating good working conditions, and solving HR problems • HR managers’ and staff experience being recognized	• leeway for crafting SHRM is perceived as good in the private hospitals, including financial instruments	• HRM practices are perceived as effective, but (mandatory) evidence is not collected • HRM practices are perceived to enable higher health service quality, but no data on patient satisfaction collected
findings for private hospitals without counterpart from public ones	• government support for international non profits	• (price) competition for patients with public hospitals which for instance provide free mother and child care	• autonomy for hospital management in relation to HRM, especially in case of non profits	• no major comparable findings	• employee engagement is perceived to positively influence employee performance and patient outcomes

Data saturation was assessed by monitoring the recurrence of themes and the depth of information obtained regarding the specific comparative questions on ownership type (PFP vs. PNFP) and contextual mechanisms. We determined that the sample size was sufficient to answer the research questions when no new major themes emerged related to the core components of the Contextual SHRM framework.

More precisely, the analysis followed the steps below, in which all authors were involved:

### Step 1

The authors familiarized themselves with the data by (re)reading transcripts and identifying the essence and patterns of meaning and potential issues of interest.

### Step 2

An initial coding scheme was developed to generate topics of interest. These initial codes were identified following a deductive coding approach based on the conceptual SHRM framework.

### Step 3

The authors verified whether the initial list of codes covered all elements of Paauwe's model and resolved any gaps in so far as possible. Moreover, the authors inductively generated open codes emerging from the data that did not appear to directly relate to the contextual SHRM framework.

### Step 4

Broader code groups were created for each theme, and subgroups of codes were created for code groups with a large number of codes.

### Step 5

All codes were combined into agreed-upon broader code groups and themes that were based on similarities and (visualized) linkages in the data and the framework.

### Step 6

The themes found were analysed and synthesized into results, as presented below.

### Step 7

We conducted a comparative analysis of the major findings from private hospitals to identify similarities and differences with results obtained for public hospitals (See Table 3 for details).

### Step 8

We elaborated the main themes (results) from private hospitals for which there were corresponding themes (results) for public hospitals (Presented in Table 3).

### Step 9

We elaborated the themes for private hospitals without counterparts from public ones.

### Step 10

Re-examined the data to prevent overlooking themes identified for public hospitals but yet without counterparts from private ones.

Ethical approval was obtained from the Ethiopian Public Health Institute (Approval NO. EPHI-IRB-131-2018).

## Results

According to the health policy (55) and strategic HRM (10, 11, 26) of Ethiopia/central government, the country maintains a pluralistic health system in which private for-profit and not-for-profit hospitals complement and supplement the public sector and HR practices. Within this mixed healthcare system, the government has a responsibility to regulate hospitals which it does through a regulatory framework that stewards the Ethiopian society and encompasses providers regardless of ownership model. Respondents perceived the framework to make few distinctions between hospitals depending on ownership type (e.g., in terms of facility licensing and accreditation, professional's certification, health service scopes, training/continuous education requirements). The regulation for instance specifies that PNFP hospitals are essentially regulated by the CSO Law (Proclamation NO.113/2019 for Charities and Societies). As not-for profit organizations, they are not paying taxes (except on staff salary). In addition to the aforementioned law, many PNFP hospitals are also governed by an NGO, e.g., by a faith-based/religious institution with its own regulations influencing SHRM (56).

The Commercial Registration and Licensing Proclamation (NO980/2016) and the related Inspection Directive (NO.528/2013) are the major government control instruments for entities with profit objectives such as PFP hospitals, and regulates their taxes, including profit taxes (57, 58).

The other national laws, regulations, legal frameworks and standardization policies of the government are context and sector specific and distinguish the three types of hospital ownership for some but not for all areas of regulation. For instance, private hospitals have more leeway (than public hospitals) regarding salaries and the provisioning of other benefits for their workforce (59–65).

### Institutional mechanisms: the strong governmental control and its impact on SHRM

In alignment with the above, a main finding regarding the institutional mechanisms' component of Paauwe's model regards the dominant role of the government, as demonstrated by the perceived tight, control-oriented regulation, which was felt to leave limited room to maneuver in crafting SHRM. Respondents from PFP hospitals gave several examples of governmental control-oriented tight regulation, such as rigid standardization policies, regulatory frameworks, and hospital reform guidelines.

“Respondents from PFP hospitals gave several examples of governmental control-oriented tight regulation, such as rigid standardization policies, regulatory frameworks, and hospital reform guidelines. In addition, the majority of respondents reported that the strong governmental regulatory control impacts crafting of SHRM.” “The tight and coercive standardization policy influences for-profit hospitals, contrary to our expectation for efficient, effective, and quality healthcare and patient outcomes(Riii).”[PFP]

“The government is playing a dominant role in strictly controlling and pressurizing us (PFP hospitals) compared to public and PNFP hospitals. There is inconsistent policy and regulatory support, as well as poor monitoring capacity of the government as a regulator. Additionally, the government’s less supportive regulations and reform guidelines require highly skilled professionals who demand high salaries from PFP institutions. The government is lacking the capacity for protective stewardship towards PFP hospitals, while providing more support to PNFP and public ones, without considering our competition for patients (Riv) [PFP]. ”Some laws (civil service reforms/regulations) are non-applicable to for-profit hospitals in terms of goals, and salary system for staff though we use government labor law for HRM as a guide (Rx).'[PFP]

By contrast, not-for-profit private hospitals reported good or flexible governmental support, with seconded professionals and budgetary top ups for hospital service improvement to achieve their goal of serving the most vulnerable and poorest communities without profit objectives.

“The hospital (missionary/international) is politically supported by the government with budget top-ups, which motivates us to strive for higher quality services for the poor community and enhances the effectiveness of our HRM practices, including staff motivation and retention (Rii)[PNFP]. Our hospital (PNFP/international) has greater autonomy to design and implement our own HRM strategy aligned with government regulations and policies (Ri).”

“…also guided by our friary regulation, which received positive feedback from the government, it enhanced our capability for efficiency, flexibility, and innovation in providing good quality care and better managing our workforce. The health ministry is also supporting us (PNFP, missionary hospital) with the secondment of professionals and free skills training opportunities (Rii).”

Although the governmental regulation is perceived strict, the tightness of implementation thus may vary in practice. Some hospitals failed to implement mandatory HR practices such as conducting annual employee satisfaction surveys and documenting staff profiles (e.g., implementing HMIs, HRIs). Among the reasons provided were capacity shortages to be able to comply with regulatory guidelines. For instance,

“Lack of regular staff surveys and the use of ICT/HRIs are mainly due to owners (PFP) being unwilling to implement them for fear of the associated expenses (e.g., the cost of IT devices and the anticipation of inquiries from staff regarding salary increases) (Rvii).”[PFP]

The private hospitals encounter several consequences of this tight regulation. As the rigid policy/regulatory framework requires highly skilled professionals, these professionals demand high salaries. The scarcity and high cost of human resources sometimes caused hospitals to struggle with providing some of the services, possibly resulting in loosing clients.

“We (PFP) are bound by a rigid regulatory framework and standardization policy that hinder our service scope in providing services [e.g., maternal and child healthcare] that are freely available to many customers in public hospitals. Our skilled professionals continuously need raises in their salaries and duty allowances, while our income from customers is shrinking (Riv).”

“We are also losing many of our clients, especially in the areas of antenatal, delivery, postnatal, maternal, and child healthcare. We ask for very low costs for clients who are not able to produce a poverty certificate from local governments for free services, as these services are provided freely at the public hospitals (Rii).”[PNFP]

“Our hospital is losing income and is unable to pay salaries for a highly skilled professional workforce because many of our patients have already left us to seek free services in public hospitals (Riii).”[PFP]

### Competitive mechanisms: private-for-profit hospitals are competing for patients

While public hospitals reported to compete for qualified professionals to be able to meet demand, private hospitals reported to mostly compete for patients. This competition for patients was importantly caused by the alternative of attending public hospitals that provide services for free or at low, subsidized, prices.

“We are unfairly competing for patients, largely due to the free healthcare services provided in public hospitals, which impact expenses for medical inputs and payments for skilled professional staff (Riv).”[PFP]

In addition, some respondents mentioned to compete for professionals, for instance in exceptional cases of scarce professionals, and when younger professionals considered to migrate to public hospitals that offered better training opportunities.

“Due to competition for patients, as well as declining profits, some professionals, including nurses and general practitioners, are dissatisfied with their working conditions here. Additionally, they are pulled and attracted to educational opportunities and on-the-job skills training offered at public facilities, making it challenging for us at PFP Primary Hospital to retain them (Rvii).”[PFP]

In such cases, private hospitals deployed innovative SHRM practices to retain staff.

“We (at PFP General Hospital) occasionally implement innovative practices involving negotiated agreements with scarce and costly health professionals, such as radiologists, cardiologists, and neurologists, for scheduled deployments. These arrangements include exceptional compensation models that allow us to allocate a significant portion of the revenue from the cost of care, as well as additional allowances. This approach has enabled us to provide efficient and high-quality healthcare (Rvi).”

“We (PNFP) innovate our HR practices, such as task shifting and sharing, by providing skills training in emergency surgery and redistributing tasks among midwifery nurses. This innovative HRM approach aims to enhance access and healthcare outcomes for fistula survivors on behalf of senior fistula surgeons. Additionally, we facilitate mentoring for former fistula survivors, who have served as administrative staff for many years, by deploying them as “junior nurses and mentors” to provide care and support to patients (Ri).”

### Heritage mechanisms: A heritage of good human resource management leeway

Not-for-profit hospitals mentioned to have a heritage of high leeway in implementing financial and non-financial HRM practices. Consequently, not-for-profit hospitals particularly reported a well-organized actual HRM strategy which positively influence workforce management. However, for-profit hospitals also mentioned a heritage of limitations in crafting SHRM practices due to the aforementioned tight government control. For instance,

“…. our SHRM strategy, guided by the existing past HR strategy and regulations, has effectively helped us attract and retain talent by valuing our staff as valuable assets. Surprisingly, we at PFP have not experienced issues with client loss; instead, we have a large customer base built on trust, loyalty, and a strong reputation. Patients believe that the quality of services at our hospital is superior to the free services provided by public hospitals (Rvi).” [PFP]

“The hospital Board and senior leadership members are competent and skilled in engaging staff and addressing HRM challenges. Supportive senior leadership recognizes staff experiences and empowers the HR department. We have an effective SHRM approach that aligns closely with the national HRM strategy, addressing key workforce needs such as wellbeing, good working conditions, financial arrangements, and non-financial incentives (Ri).” [PNFP]

“Our friary”s regulation and administration system, which has received political and governmental support, has created conducive working conditions. It possesses HRM autonomy and fosters a climate that enables the development and implementation of effective HR policies and practices that view employees as valuable assets. Furthermore, this system is well aligned with the organizational goals and structures (Rix).’ [PNFP]

All studied private hospitals report they are respecting legislative compliance, yet the reality of for-profit hospital legitimacy compromised due to full dependence on owners' good will and decision.

“Leadership capability is linked to the owners” wise decision to pay high salaries for skilled professionals, as well as to the extrinsic motivation, engagement, and recognition of staff. We believe this strategy or approach helps us meet patients' needs by providing timely and quality healthcare. However, we do not assess satisfaction surveys, as this requires the goodwill of hospital owners due to concerns that unfavorable outcomes might lead to additional costs or bonuses for staff (Rviii).' [PFP]

“Our hospital leadership has fostered a supportive working environment for HR managers and staff that effectively addresses HR challenges. We, at PFP, offer competitive wages and duty allowances while actively engaging and motivating all staff through bank loan arrangements for purchasing homes and vehicles. Additionally, we have no reported incidents of moonlighting (Rvi).” [PFP]

The administrative leeway and more flexibility regarding bundles of HRM practices in the private (PNFP) hospitals mainly related to friary regulation and a bundle as a way to work around the legislation stimulates workforce to provide client-centered high quality health services. For example,

“Due to greater autonomy related to HRM leeway, we (PNFP) are successfully attracting and retaining best talent by recognizing the roles of HR managers and prioritizing staff wellbeing. We provide comprehensive medical insurance coverage and offer free lunch and breakfast for all employees. Additionally, we have implemented innovative practices such as task shifting, which includes light training and mentoring for over 198 ex-fistula victims/survivors, empowering them to take on roles as “nurses”. We also offer flexible working hour arrangements with duty allowances and hire retired expert professionals to address specific skill shortages or gaps (Ri).”

### Leeway for crafting SHRM

The leeway was found to be conducive to implementing innovative, self-initiated, bundles of SHRM practices aligned to the central government HRM strategy. These HR practices entail motivation enhancing practices (sometimes combining financial and non-financial incentives), as well as ability- and opportunity-enhancing practices of facilitating on-the-job skills training for mid-level professionals.

“We are implementing non-financial incentive packages aimed at enhancing employee wellbeing. These include arranging free lunch and breakfast services, providing medical insurance, motivating staff, and recognizing team achievements (Ri).” [PNFP]

“Facilitating annual salary increments, providing scholarships for high performers to attend training, offering competitive salaries and allowances, covering transportation costs for travel, and arranging loans for purchasing homes, are all practices that help improve employee performance, ensure efficiency, and deliver high-quality health services in our[PFP] hospitals (Rvi).” [PFP]

“We have a well-established organizational structure that gives space and allows HR managers the autonomy to design and implement our own SHRM systems or policies/strategies (Rii)” [PNFP].

“ HR managers are empowered to make decisions on all HR management issues, effectively managing our engaged workforce, which positively impacts HRM outcomes (Ri).” [PNFP]

“Our employees are actively involved and engaged in decision-making on all hospital management issues, including setting incentives. This positive involvement results from our HRM strategy, which is rooted in the national SHRM strategy and particularly informed by the friary regulation that allows for these unique or innovative HR practices (Ri).” [PNFP]

“Our hospital”s teamwork culture, staff engagement, motivation, and promotion practices positively influence workforce motivation and act as a driving force for delivering efficient, high-quality, and free/subsidized or affordable healthcare services (Rii).” [PNFP]

### SHRM system, HRM outcomes and influence on performance

Our respondents suggested that their SHRM practices positively influenced HRM outcomes and patient outcomes including quality of services in Ethiopian private hospitals. However, none were able to provide data to support such claims, nor for patient satisfaction, nor for employee satisfaction, as should be collected through mandatory annual surveys.

“Even without satisfaction survey data, our exit discussions with customers indicate that our PFP patients are satisfied with the high-quality health services provided by our engaged staff, particularly in nursing care. Our hospital is implementing flexible and innovative HRM practices, such as involving skilled professionals, providing annual salary increments, engaging staff in holiday duty assignments, and incorporating teaching opportunities in our college, all of which stimulate and boost their performance. This level of engagement is also evident in staff satisfaction, as reflected in our annual staff meeting (Rviii).”[PFP]

“Our hospital is implementing a flexible SHRM system that motivates our professionals by offering competitive salaries and allowances. Additionally, we encourage senior professionals to engage in teaching and research at our college. In my view, the positive outcome of having highly paid professionals and an engaged staff is that they are dedicated and passionate about delivering customer-centered, high-quality, and efficient support and services (Rix).” [PNFP]

Table 3 summarizes the main findings per framework component, emphasizing the differences between the hospital types.

## Discussion

Despite the limited sample size, observable descriptive patterns emerged: PNFP hospitals reported greater autonomy and government support, while PFP hospitals emphasized competition for patients and reliance on financial incentives.

Guided by the contextual SHRM framework (49), we conducted qualitative research based on document analysis and interviews to better comprehend how contextual (institutional-, competitive- and heritage) mechanisms influence strategic HRM and performance in private hospitals in Ethiopia. We also conducted a SHRM-wise comparison to understand how the corresponding mechanisms impacting SHRM practices and performance in private hospitals differ from the mechanisms in public hospitals. The study considered that the combination of these contextual mechanisms importantly shapes SHRM in the studied private hospitals. Specifically, institutional mechanisms constrain SHRM autonomy, competitive mechanisms drive differentiation strategies, heritage mechanisms influence organizational flexibility, and leeway enables innovative HR practices.

In Ethiopia, the government regulates public and private hospitals through policies and health system and regulatory frameworks that serve the public interest. Our previous studies revealed that public hospitals perceive these regulations to restrict SHRM autonomy including fair pay and better working conditions, perceived as among the causes of nationwide strikes by healthcare professionals in the state-owned hospitals. These hospitals experience coercive pressures and are often constrained by a legacy of limited HR management flexibility, which is regarded as a long-lasting heritage factor (10, 11). PFP hospitals also see the regulation as strict and overly rigid. A main finding, however, is that PNFP hospitals perceive the policy and regulatory frameworks as providing beneficial flexibility and support.

PNFP hospitals are primarily owned by international organizations, fostering a distinct relationship with the government, which can be characterized as a constructive engagement. Focusing on providing affordable health services to underserved communities, the PNFP hospitals report to receive policy/political and budgetary support to improve health service provisions. The SHRM practices, especially of the PNFP hospitals who received special government support (e.g., skills training opportunities and secondment of professionals), have enabled PNFP hospitals to help address the capacity shortages of the public sector hospitals. This finding is in line with previous studies in the African and related (PNFP, PFP) contexts (12, 31, 36, 66–71) that have highlighted the influence of institutional mechanisms/government role and policy reforms to address SHRM challenges.

In contrast to the results for PNFP hospitals, PFP hospitals perceive regulations as rigid and control to be tight. At the same time, we find that they fail to fully utilize the available leeway for SHRM and often adhere more passively to legacy government standards than necessary. As a result, they appear to be overly impacted by the pressure to comply with government-mandated standardization and legal frameworks. PFP hospitals could, for instance, take more advantage of the leeway to adopt (HR bundles that include) non-financial instruments to enhance employee well-being. Our findings thus align with previous studies reporting that PFP hospitals appear more inclined to apply financial incentives to motivate staff than other ownership types (7, 10, 28, 70–74).

While our findings suggest that PFP hospitals significantly shape their SHRM practices according to reform guidelines and regulatory requirements, many private hospitals either partially comply or completely disregard governmental regulatory mandates in case of inconsistencies or weak implementation. For instance, weak enforcement of policy/regulatory frameworks by the government coupled with a lack of interest from hospital owners often leads to non-compliance with mandatory annual employee surveys in PFP hospitals as required by the human resource data management regulations. This contrasts with findings for PNFP hospitals, which additionally adhere to international SHRM standards while viewing government role of regulations as beneficial. Drawing on the SHRM model, we may notice that the PNFP hospitals perceive greater formal “leeway” and “room to maneuver”, whereas the PFP capture some leeway which is formally doesn't exist (49).

The synthesis of our findings demonstrates that institutional mechanisms act as the primary driver of SHRM strategies. For PFP hospitals, rigid regulatory frameworks restrict formal leeway, forcing reactive SHRM that relies heavily on financial compensation to attract scarce talent in a competitive labor market. Conversely, PNFP hospitals benefit from a “constructive engagement” as institutional mechanism, granting them the leeway to adopt mission-driven and innovative SHRM practices that align with non-profit objectives. This interplay between institutional pressure and available leeway directly shapes SHRM strategies and outcomes; PFP hospitals focus on competitive positioning through service quality (to justify higher prices), while PNFP hospitals focus on mission fulfilment and addressing underserved populations.

Our study illuminates how the SHRM of PFP hospitals is built around employing highly skilled workforce as a means towards delivering higher quality, timely and more valuable hospital services to justify the higher price charged to patients. SHRM thus forms a critical element of creating a competitive advantage through a differentiated value proposition (75, 76). The higher charges cover the cost of more attractive financial employment conditions which helps PFP hospitals to resolve the difficulties to attract skilled professionals experienced especially by public hospitals. Thus, we find that for PFP hospitals, competition is primarily for patients. This contrasts with findings for public and PNFP hospitals which are confronted with an abundance of patients yet compete for health human resources. However, the healthcare services provided for free or at very low charges by these public and PNFP hospitals cause price and costs pressures for PFP hospitals that require them to balance efficiency with perceived quality, prompting SHRM challenges for which they are still learning to properly and fully utilize the available leeway (75, 77–79).

Theoretically, this study extends the application of Paauwe's Contextual SHRM Framework by validating its relevance in the private sector of a low-income country. It confirms that institutional mechanisms (government regulation) do not impact hospitals uniformly; instead, their effect is moderated by ownership type (PFP vs. PNFP). This finding contributes to the SHRM literature by highlighting the importance of disentangling “private sector” strategies, revealing that profit motives vs. social missions lead to fundamentally different responses to the same external regulatory environment.

### Strengths and limitations of the study

This study includes a varied sample of Ethiopian PNFP and PFP hospitals covering various specialties, geographic locations, rural and urban settings, and central and regional governments. While we included diverse hospital levels and locations, our analysis indicates that ownership type (PFP vs. PNFP) was the primary driver of differences in SHRM perceptions and challenges. We observed consistent institutional pressures and leeway across different hospital levels and geographic settings, largely due to the uniform application of national regulatory frameworks. Furthermore, as data were collected between March and September 2019, the findings should be interpreted as a reflection of the structural and regulatory dynamics at that time. The Ethiopian health sector has since undergone significant shifts, including the impacts of the COVID-19 pandemic and internal conflicts. While the fundamental institutional mechanisms described are likely to persist, some specific pressures and competitive dynamics may have slightly evolved in response to these macro-level changes. Hence, the current situation is not significantly different, especially from the context of the themes we are addressing and role of the government are still remain similar.

The study particularly engages various respondents, ranging from experienced hospital administrators, operations managers and HR managers to matrons, team leaders and professionals. This triangulation within interviews was further strengthened by document analysis and an in-depth comparison with companion qualitative data for public hospitals. Previous studies were largely based on secondary data and gave little attention to external factors and hospital ownership types when studying strategic HRM in Ethiopia and Sub-Saharan Africa (e.g. (10, 11, 28),.

A first limitation of our study concerns the regional conflicts within Ethiopia that restricted travel to study settings in some regions (Oromia, SNNP) and caused delays in data collection and analysis. The variety in circumstances in the large, diverse and dynamic federal state Ethiopia have also impacted the data collection opportunities. As a result, we cannot ascertain saturation and may have missed some factors, potentially limiting validity of our findings for regions not included in the data collection. Saturation in a country as large, dynamic, and complex as Ethiopia may be near impossible. Second, the collection of data from private hospitals included HR managers and administrators but did not involve employees. *Hence, a* key limitation of this study is that the findings rely on the perspectives of hospital leadership, including CEOs, administrators, and HR managers. The views of medical professionals and employees were not captured, which may limit the validity of the claims regarding HRM effectiveness, staff satisfaction, and patient outcomes. Future research should incorporate employee perspectives to provide a more holistic view of SHRM impact. Therefore, inclusion of such respondents is recommended in future research (see, e.g. (14–17, 43),. Third, the study focused on private hospitals in Ethiopia and findings are strongly impacted by the Ethiopian policies and regulations. Therefore, the generalizability to other healthcare contexts may be limited.

## Conclusions

This study makes both theoretical and empirical contributions to the field of Strategic Human Resource Management (SHRM) in low-resource settings. Theoretically, it advances the Contextual SHRM Framework by demonstrating how the interplay between institutional pressures and organizational heritage varies significantly across private ownership types. Empirically, it provides rare insights into the SHRM practices of the Ethiopian private health sector, explicitly contrasting the constraint-driven strategies of private-for-profit hospitals with the supportive, mission-driven strategies of private not-for-profit hospitals. This extends existing research, which has predominantly focused on public hospitals, by highlighting the unique competitive and regulatory dynamics faced by private providers.

Our study reveals that stringent governmental policies and regulatory frameworks constrain the SHRM of PFP hospitals, often with excessive impact. Conversely, when weak regulatory enforcement is weak, PFP hospitals exploit leeway that formally is not available. These PFP results contrast with the results obtained for PNFP hospital that perceive regulation as supportive. This situation differs from public hospitals, which lack the leeway for crafting their SHRM. While our findings indicate a positive link between SHRM practices and improved HR and patient outcomes in Ethiopian public hospitals, the lack of empirical data inhibits investigating this relationship for private hospitals. Regulatory compliance and future research can thus shed further light on the effectiveness of SHRM in the private hospitals.

Accordingly, our analysis leads to the following recommendations:

### Recommendations for practice

- To enhance the contribution of private hospitals, the government can foster collaborative mechanisms and public-private partnerships. This includes creating an incentivized regulatory framework that acknowledges the contributions of private hospitals without negatively impacting workforce requirements of the public sector serving the far majority of the population.
- Despite the rapid expansion of the private hospital sector, compliance with SHRM standards remains inconsistent. The government should develop targeted strategies to enhance regulatory capacities and effectively oversee the sector.
- PFP hospitals can better utilize the SHRM leeway policy and regulatory frameworks offer and foster the relationship with government by complying with existing frameworks.
- Our findings suggest that the private sector hospital HRM and performance can benefit from policies that offer more HRM leeway, especially for policies for which enforcement capacity is lacking.

### Future research

- Engaging with hospital employees in future studies will provide valuable insights into SHRM practices and performance concerning hospital ownership structures, addressing a significant gap in existing literature.
- Extend the research to include employee and patient outcomes to enable assessment of SHRM effectiveness.

## Data Availability

The original contributions presented in the study are included in the article/Supplementary Material, further inquiries can be directed to the corresponding author.

## Footnotes

^1^PNP, invests any net income remaining after accounting for all expenses into the same institution in pursuit of its social mission [5,25,46]. This is different from PFP hospital for which any financial margin after expenses may go to the owners [47].
