# Supplementary material for: The role of context in strategic human resource management within private hospitals in Ethiopia, compared to public hospitals
Source: Front Health Serv. 2026 Feb 19;6:1740353. doi: 10.3389/frhs.2026.1740353 (PMC12960097; doi:10.3389/frhs.2026.1740353)
Supplement: Supplementary file 1 [file Table1.docx]

**Supplementary materials (Appendix 1 and 2)**

**Appendix 1 (Supplementary data) : Topic list and interview guide to study SHRM in Ethiopian Hospitals**

Dear Sir/Madam;

To introduce myself and aim of the study, I am …… conducting research on HRM practices in Ethiopian hospitals. The aim of the study is to hear your experiences and opinion so as to advance our understanding on how HRM strategies evolve in the Ethiopian private and public hospitals setting. To this end, I would like to interview you, and thank you in advance for your cooperation in sparing your busy schedule.

I would like to ask you questions about several topics that relate how HR strategy is developed in this hospital. There are no good or wrong answers to the questions. This interview will approximately take 75 minutes of your time. We are asking your permission to audiotape the interview (verbal consent), which do not aim to identify and describe your name, personality but only for research purpose. Indeed, your responses will be kept strictly confidential.

1. **Background and introduction questions:**
   1. Could you tell me something about your job and responsibilities within this hospital?
   2. What is your educational background and experiences in this and similar organizations?
   3. How long do you work in this hospital, in the current position?
   4. What is your relationship to the HR strategy? (possible answers: responsible to develop it, responsible to implement it, ...)
2. **Institutional Mechanisms[from health system, regulation & legal, political, cultural & societal contexts)**

2.1. Which characteristics and developments in the health system impact the way you manage the hospital and especially your employees (professionals and staff)? Think for example of hiring, salary, working hours and conditions,…..

2.2. Which governmental regulations and laws impact the way you manage the hospital and its workforce? (e.g. national labor laws and regulations, regulation for healthcare institutions)

2.3. What political aspects are influential on your management of the hospital and especially of your employees? Could be your direct relationships, or the relationships of your institution, e.g. with Health Bureau, University, Ministry, Politicians,…

2.4.How do societal and cultural characteristics and developments impact your management of the hospital and especially of the employees ? Think for example on (changing) demand and expectations, economic developments, poverty, health literacy,**…**

1. **Heritage Mechanisms related questions:**

3.1. Can you sketch the organizational structure? Is there a dedicated HR department? Where is it positioned?

3.2. What responsibilities are with the HR department? What HR responsibilities are with executive management? What with the line management?

3.3. What is the history of this hospital, especially the recent history? What is special? What are the strengths, especially in workforce, skills, knowledge. What are challenges in HRM, workforce, skills, knowledge,….

3.4. How would you describe the organizational culture?

1. **Questions related to Competitive Mechanisms :**
   1. What services do you offer? What do you offer especially? What is it that you don’t offer?
   2. Can you describe the market, i.e. your customers and your competitors?
   3. What are the main developments and innovations in the market? Which technologies matter?
   4. Which skills are important? Are you competing for skilled personal? Do they leave for the competitor?
2. **Questions related to ‘Organizational capabilities’ related factors :**
   1. Do you achieve the desired health outcomes for your patients (are you effective)?
   2. Are you responsive? Can you instantly address urgent patients? Do you have short waiting times?
   3. Are you efficient? Do you have low cost? OR are you rather focused on being high quality for instance?

5.4. Do you learn new technology and procedures easily? Can you innovate?

5.5. Do you manage to be compliant with all regulations on hospital care? Where are the challenges? How is it related to human resource management?

5.6. Do you manage to be compliant with all regulations for labor / employee relations? Where are the challenges?

5.7. Can you get all work done within the skills and time of the corresponding employees?

5.8. Can you divide work and manage employees equally and fairly?

5.9.Are employees involved in decision making? Do you think they feel engaged? Involved?

1. **Questions related to Leeway**
   1. Who are the key decision makers in general hospital management?
   2. Who decides on HRM (strategy)?
   3. Could you tell me about your mandate/authority?
   4. Who decides on job description? Who on salary system and salary scale for health professionals? On salaries, promotions, allowances, incentives? Moonlighting? Hiring/firing?

6.5. Is HRM important for your hospital and why?

6.6. What are the roles/functions of CEO or General Manager of this hospital?

6.7. If you would like to change the present HR strategy, would this be possible? To what extent? Who would be able to alter HRM? Where can you advance? Where are you bounded?

6.8. What promotes, what hinders the responsibilities of decision maker/s in this hospital?

6.9.Which factors influence the choices and implementation of HR strategy?

**Could you provide additional documents** about this hospital [establishment, organogram, bed size, staff size, HRM policy/strategy/guideline, Hospital Management Committee/Board profile, annual patient load, job- and patient- satisfaction, recognition award (if any), other KPIs reports, …]?

**Abbreviation:** MOH: Ministry of Health; FMHACA**:** Food, Medicine and Healthcare Administration and Control Authority; CRC: Compassionate, Respectful and Caring; KPI: Key Performance Indicators

**Thanks a lot for your time and cooperation**!

**Participant Information Sheet (PIS) and Consent Form(CF)**

***Dear madam/sir,***

Below, I wish to briefly introduce you why this study is being done and what it will involve.

To introduce myself and aim of the study, I am …. conducting research on SHRM practices in Ethiopian hospitals, fully funded by Erasmus University Rotterdam and expected to be completed in April 2019. The aim of the study is to hear your experiences and opinion so as to advance our understanding on how HRM strategies evolve in the Ethiopian hospitals setting. To this end, I and the research team member would like to conduct group discussion, and thank you in advance for your cooperation in sparing your busy schedule. I would like to ask you questions/professional experiences about several topics that relate how HR strategy is developed in your and/or other hospital. There are no good or wrong reflections/answers to the questions. This discussion will approximately take 90 minutes of your time. We are asking your permission to audiotape the group discussion, which do not aim to identify and describe your name, personality but only for research purpose. Tapes will be identified only by a code, and will not be used or made available for any purposes other than the research project. These tapes will be destroyed at the end of the study. *Y*our active participation is instrumental and your responses will be kept strictly confidential.

You have been chosen and approached because of your experiences and roles associated to this research and societal implications to hospitals and health system. Taking part is entirely voluntary, beneficial to improve societal health, HRM in hospitals and organizational performance. There is no intended personal benefit (but social) in taking part, has no risks and that refusal or withdrawal will involve no penalty or loss, now or in the future but your information is vital. Thus, I kindly expect you will give genuine information, experiences, ideas or opinions. We would be happy to share a copy of (if published) results where you will not be identified in any report or publication. The project has received ethical approval certificate from the Ethiopian Public Health Institute (EPHI) of the Federal Ministry of Health*.*

**Consent Form**

- I have been debriefed, read and understood the Participant Information Sheet;
- I have been given the opportunity to ask questions and have had them answered to my satisfaction;
- I agree to take part in this research project;
- I understand that my participation is voluntary and I have been informed that I am free to withdraw at any time without giving a reason;
- I understand and agree to consent for EUR and scientific procedures for handling any personal data (e.g. confidentiality, anonymisation, etc.);
- I understand and agree to consent to proposals for data storage, archiving, sharing and re-use for future research;
- I understand and agree to consent to any planned audio or visual recording.

I kindly ask you to sign, print your name and date this form

| **Print your name** | **Signature** | **Date (GC)** |
| --- | --- | --- |
|  |  |  |

**Appendix 2. Human Resources Management Implementation Checklist with scope of practices**

| Standard HRM practices required to be implemented in hospitals | Yes | No |
| --- | --- | --- |
| Policies and procedures for staff recruitment, promotion and transfer have been developed and implemented |  |  |
| Policies and procedures for performance evaluation have been developed and implemented. |  |  |
| Policies and procedures for employee recognition have been developed and implemented | . |  |
| Policies and procedures for training and development have been developed and implemented. |  |  |
| Policies and procedures for compensation and benefits have been developed and implemented. |  |  |
| Policies and procedures for occupational health and safety services have been developed and implemented. |  |  |
| Policies that define when and what type of identification badges and uniforms are worn by staff have been developed and implemented. |  |  |
| A Human Resource plan has been developed |  |  |
| Training Need Assessment has been conducted and Training and Development Plan developed |  |  |
| Adequate budget is allocated for Human Resource Management/Development |  |  |
| The Human Resource head is represented on the Senior Management Team. |  |  |
| Adequate HR professionals are assigned to provide human resources management services |  |  |
| Job descriptions have been developed for each position at the hospital |  |  |
| Staff job satisfaction survey is conducted regularly. |  |  |
| HR Audit has been conducted |  |  |
| Each employee has a personnel file that is maintained by the Human resource directorate/department. |  |  |
| A Functional Human Resource Information Management System (HRIS) has been established |  |  |

Source: Document Analysis of the Ethiopian Hospital Services Transformation **Guideline**, Chapter 17: HRM Section, and Ethiopian Hospital Reform Implementation Guideline, Federal Ministry of Health, 2010[MOH, 2018]
